# Transient Induced Molecular Electronic Spectroscopy (TIMES) for study of protein-ligand interactions

**DOI:** 10.1038/srep35570

**Published:** 2016-10-19

**Authors:** Tiantian Zhang, Ti-Hsuan Ku, Yuanyuan Han, Ramkumar Subramanian, Iftikhar Ahmad Niaz, Hua Luo, Derrick Chang, Jian-Jang Huang, Yu-Hwa Lo

**Affiliations:** 1 Materials Science and Engineering Program, University of California San Diego, La Jolla, California 92093-0418, USA; 2 Department of Electrical and Computer Engineering, University of California San Diego, La Jolla, California 92093-0407, USA; 3 College of Basic Medicine and Forensic Medicine, College of Manufacturing Science and Engineering, Sichuan University, Chengdu, Sichuan 610041, China; 4 Department of Nanoengineering, University of California San Diego, La Jolla, California, 92093-0448, USA; 5 Graduate Institute of Photonics and Optoelectronics, National Taiwan University, Taipei, 10617, Taiwan; 6 Department of Electrical Engineering, National Taiwan University, Taipei, 10617, Taiwan

## Abstract

We present a method, Transient Induced Molecular Electronic Spectroscopy (TIMES), to detect protein-ligand interactions without any protein engineering or chemical modification. We developed a physics model for the TIMES signal and mathematically formulated the problem to attain physical insight of protein-ligand interactions without any disturbances by molecular probes, fluorescent labels, or immobilization of molecules. To demonstrate the functionality of this method, we have used the TIMES signals to find the dissociation constants for the affinity of reactions, the shear-stress dependent adsorption time of molecules on surface, and other interesting features of protein-ligand interaction in native conditions. As a unique tool, TIMES offers a simple and effective method to investigate fundamental protein chemistry and drug discoveries.

Protein-ligand interaction plays the central role in biomedical process and drug discovery^1^,^2^. While computer simulations^3^ and high-throughput screening methods^4^,^5^ have been widely applied to perform early stage screening of drug candidates, limited method is available to investigate the effect of protein-ligand interaction without any external disruptions. There have been several sensing techniques for investigation of protein-ligand interactions, including surface plasmon resonance (SPR)^6^,^7^, isothermal calorimetry (ITC)^8^, biologically modified field effect transistors (BioFET)^9^, differential light scattering (DLS)^10^, fluorescence resonance energy transfer (FRET)^11^, electrophoretic mobility shift (EMSA)^12^,^13^, and small molecule microarray^4^, etc. Most of these methods can measure binding affinity, kinetics, and other thermodynamic characteristics of protein-ligand interactions. However, there are still open and important problems not addressed by the existing methods: (i) Using fluorescent labeling on biomolecules in FRET, EMSA, and small molecules microarray detection methods, external modifications are added to the molecules, which could affect the binding sites or molecular structural configurations. (ii) Using surface immobilization in SPR and BioFET techniques, spatial limitation is introduced to alter the entropy of the system, which can affect the experimental results by limit protein movements or protein folding/unfolding, and cause discrepancies from reactions in physiological conditions. (iii) Techniques such as ITC relies on heat release from the reactions have relatively low resolution, produced limited information on reaction kinetics, and face difficulties in reactions that do not generate a large amount of heat (e.g. entropy driven rather than enthalpy driven reactions). (iv) Optical methods such as DLS only work for proteins that can crystalize or produce aggregation, with other constraints on the critical temperature and concentration.

Here we report a method, Transient Induced Molecular Electronic Spectroscopy (TIMES), to detect protein-ligand binding without the above constraints. The TIMES method measures the signal caused by the dipole moment change when protein and ligand form protein-ligand complex, breaking new grounds for studies of protein-ligand interaction. The TIMES signal has an excellent signal-to-noise ratio and timing resolution even though the difference in the molecular weight and chemical composition between protein and protein-ligand complex could be very small, sometimes less than 1%. The TIMES method produces signals related to the dipole moment and charge distribution of biomolecules, thus providing not only undisturbed signal in physiological conditions but also signals revealing molecular properties unattainable by and complementary with the existing methods including FRET, SPR, etc. We report some key characteristics and attractive functions of the TIMES signals, including measurements of reaction dissociation constants between proteins and ligands.

To create a flux of protein molecules towards the electrode, we designed a microfluidic device to produce a concentration gradient along the height of the channel. In our TIMES setup (Fig. 1a), the microfluidic channel has two inlets, one for the buffer solution and the other for introducing the molecule of interest (i.e. protein molecule or mixtures of protein and ligand) and one outlet. The entire channel was at first filled up with the buffer solution and then the molecule of interest was introduced from another inlet (Fig. 1b). For a laminar flow^14^ the travel speed at the center of the channel is the greatest and approaches zero at the channel wall where the gold electrode is located^15^ (Fig. 1c). As a result, a concentration gradient between the center of the channel (having the highest and constant molecular concentration) and the electrode surface was established. Such concentration gradient produced a diffusion flux for the molecules towards the electrode to produce the TIMES signal.

Along the microfluidic channel there are gold electrodes connected to an external amplifier circuit. A molecule carrying a dipole moment in the buffer solution can interact with the electric field near the solution/electrode interface within the Debye length^16^, which is in the order of 1 nm for typical ionic strength. The interfacial electric field, approximately equal to the Zeta potential^17^ divided by the Debye length according to the double-layer model^18^ can “orient” the molecule according to its charge state and dipole moment to minimize the free energy. As the molecules are oriented by the surface field, an induced dipole develops due to the mirror effect of the metallic surface. In the near field condition, dipole moment of a macromolecule such as protein has the dominant effect over the net charge of the molecule, and the alignment of the dipole moment with the surface field produces a charge transfer between the gold electrode contacting the fluid and the measurement circuit system in Fig. 1c. Such charge transfer induces an electric current that is amplified and converted into a voltage signal by a transimpedance amplifier (TIA). After converting the analog signal to digital signal through an analog-to-digital converter (ADC), one can record the real-time signal originated by the field-induced dipole orientation of the molecules. In the test system, protein is the only macromolecule that possesses a large dipole moment (Supplementary Table S1), and all other ions in the buffer move around the protein to minimize the free energy of the system. Hence the detected signal is primarily produced by protein or protein-ligand complex near the electrode. Although in many cases the ligand molecular weight could be significantly less than the molecular weight of protein, the formation of protein-ligand complex can alter the 3D configuration of the protein molecule, thus changing the dipole moment and charge distribution appreciably^19^,^20^. The TIMES method monitors the native protein ligand interactions requiring no immobilization or labelling and with high temporal resolution. Next we depict the proposed physical principle that produces the TIMES signal.

We assume that with sufficient ionic strength in the buffer, the “local charge neutrality” condition is satisfied when protein travels in solution without an external field (Fig. 1d,i). Local charge neutrality assumes that charges on the surface of protein are neutralized by the mobile counter charges in the buffer within a time scale of “dielectric relation time” equal to ε/σ where ε and σ are the permittivity and conductivity^21^ of the buffer solution. Since the dielectric relaxation time is typically 1 to 100 ns, much shorter than the time scale of interest in our measurement, the local charge neutrality condition can be satisfied everywhere in the solution (Fig. 1d) except for regions right next to the electrode/liquid interface where the electric field is present. When the protein molecules approach the electrode and experience the electric field from the Zeta potential, two things take place: at first those mobile ions around the protein to maintain local charge neutrality are stripped off by the E-field and find their new equilibrium distributions, and secondly the protein molecules are oriented to have their dipole moment aligned to the direction of the field (Fig. 1d,i). The alignment of dipole moment of protein gives rise to an induced charge flow between the electrode and the amplifier input, giving rise to a detectable signal (Fig. 1d,ii). Driven by the shear stress of the laminar flow in the microfluidic channel, the protein molecules may not permanently be adsorbed to the electrode surface but detached from the gold electrode after some period of time. The departing molecules carry neighboring ions with them via electrostatic interactions and drag force, disturbing the local ion distribution and generating an overshoot in the electric signal (Fig. 1d,iii). Eventually, those ions carried by the departing proteins return to the electrode surface and the steady state is restored (Fig. 1d,iv). The TIMES signal produced by 500 nM thermolysin molecules is shown in Fig. 1e. Similar analysis and model of protein-surface interaction has been reported before^22^,^23^. The theory and mathematical formulation of TIMES signal is described next.

We assume *q*(*t*) is the induced charge in the gold electrode in response to a protein molecule reaching the electrode surface. *q*(*t*) can be treated as the “impulse response” or the “Green’s function” generated by a single protein (or protein/ligand complex) molecule approaching the electrode, having the unit of “Coulomb”. The net charge signal on the electrode, induced by all the protein molecules at a specific time, can be represented as


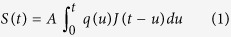


where *A* is the area of the electrode and *J*(*t*) is the flux of the protein molecules at the electrode. The flux of molecule can be represented as


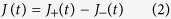


where *J*_+_ and *J*_−_ are the flux of molecular adsorption and desorption, respectively.

Next we need to find the expression for the protein concentration immediately next to the electrode surface, *n*_*i*_(*t*), which is related to the protein concentration outside the Debye length where the electric field is nearly zero:


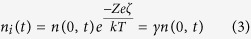


where *Ze* is the charge of the protein and *ζ* is the zeta potential. Throughout the analysis, we assume that the protein concentration is low enough not to change the ionic strength of the buffer. Therefore, the zeta potential is not changed significantly by the protein so *γ* can be treated as a constant and its value is determined by the electrode material and the buffer solution.

The transport of protein across the channel thickness is governed by the equation: 
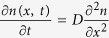
 with the boundary conditions: *n*(*x* = *L*, *t*) = *n*_*o*_ and 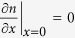 where *x* = *L* is the center of the microfluidic channel (i.e. the channel height is *2L*). We can obtain the analytical solution for *n*(0, *t*) at the position just outside the Debye length (i.e. E-field is nearly zero) as


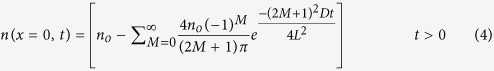


The detailed derivation is shown in Supplementary Materials.

Note that 
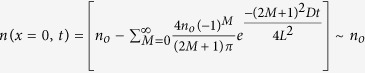
 if the time of interest is greater than 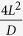 where D is the diffusivity of protein (or protein-ligand complex).

In equation (2),


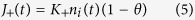


*K*_+_ has the unit of velocity, representing the rate of protein adsorption to the electrode surface.

*θ*: *fraction of monolayer deposition* 0 ≤ *θ* ≤ 1.


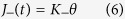


*K*_−_ has the unit of flux (1/s-area), representing the rate of protein desorption from the electrode surface.


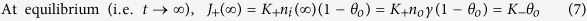


Using the relations in Eqs 5–7, we have


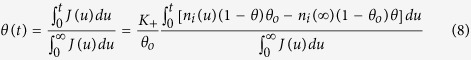


Under the approximation that the time scale of interest is significantly longer than the diffusion time (i.e. 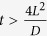), Eq. (8) can be approximated as


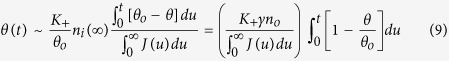


And we obtain the induced charge on the electrode by a protein molecule as


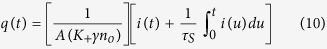



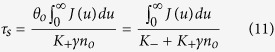


where *n*_*o*_ is the protein concentration at equilibrium, and *τ*_*s*_ has the physical meaning of the average dwelling time or surface adsorption time for a protein (or protein-ligand complex) molecule on the surface of electrode.

From (10), we show that the measured transient induced molecular current, *i*(*t*), can be used to find (a) the induced charge, *q*(*t*), by each biomolecule when approaching the electrode surface and (b) the surface adsorption time (*τ*_*s*_) of the molecule on the electrode surface without any molecular labeling or surface immobilization.

Next we demonstrate the concepts using a protein-ligand pair as example. Thermolysin is a 34.6 KD thermostable metalloproteinase produced by the Gram-positive bacteria bacteria Bacillus thermoproteolyticus. It preferentially cleaves at the N-terminus of the peptide bonds containing hydrophobic residues such as leucine, isoleucine, and phenylalanine. Phosphoramidon was isolated from Streptomyces tanashiensi, which inhibits thermolysin specifically^24^. The normalized induced charge, *q*(*t*), by thermolysin before and after binding with phosphoramidon under different flow rate is shown in Fig. 2a,b. The shear stress dependent surface adsorption time of thermolysin before and after binding with phosphoramidon is compared in Fig. 2c.

From Fig. 2a, the surface adsorption time of thermolysin decreases monotonically with increasing flow rate because a higher flow rate in the microfluidic channel produces greater shear stress according to Poiseuille’s Law^25^ to remove protein from the electrode surface, thus reducing the average adsorption time of protein molecules on the electrode. The same trend was observed for thermolysin/phosphoramidon complex (Fig. 2b). However, thermolysin and thermolysin/phosphoramidon show different adsorption time dependence on the flow induced shear stress, as shown in Fig. 2c. The results provide clear evidences that in spite of very similar molecular weight and size of the protein and protein/ligand complex, the binding strength of the two molecules to the electrode show appreciable differences, manifested in the adsorption times measured from the TIMES signals.

Under the condition that the system consists of more than one type of molecules (e.g. coexistence of protein, ligand, and protein-ligand complex), then the measured TIMES signal can be further approximated by (12) when the second term in (10) becomes negligible. This approximation is valid when the time of concern is shorter than the protein adsorption time *τ*_*s*_.


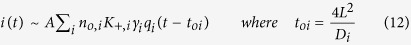


In the case of first-order reaction: *Ligand* + *Protein* ↔ *PLcomplex*


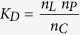


where *n*_*L*_, *n*_*P*_, *n*_*C*_ represent the equilibrium concentration of ligand, protein, and protein-ligand complex, respectively.

Assuming all *t*_*oi*_’s are short compared to the timing resolution of the measurement system, (12) can be approximated as


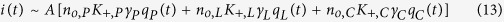


Before reaction, the initial protein and ligand concentrations are assumed to be x and y, respectively. After the equilibrium is reached, we have


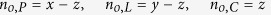


Substituting these relations into (13), we have


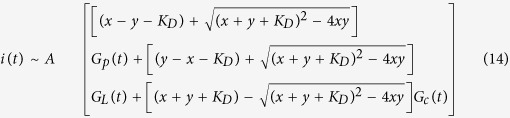


In (14) we measure the TIMES signal *i*(*t*) under given values of x and y from different mixtures of protein and ligand. Since there are four unknowns: *K*_*D*_, *G*_*p*_(*t*), *G*_*L*_(*t*), *G*_*C*_(*t*) in (14), we need to measure TIMES signals in 4 different combinations of protein ligand concentrations (e.g. protein only, ligand only, 1:2 and 2:1 protein/ligand ratios) to solve these unknowns. Among the four unknowns, only *K*_*D*_ is time independent, hence ideally we should obtain the same value of K_D_ at each time point when we solve *K*_*D*_, *G*_*p*_(*t*), *G*_*L*_(*t*), *G*_*C*_(*t*). However, when noise is added to (14) as a random variable, it causes fluctuation of *K*_*D*_ obtained at each time point. At a sampling rate of 1 ms over 1 s period, we obtain 1000 values of *K*_*D*_. Therefore a histogram of *K*_*D*_ value can be generated to help us determine its mean value to minimize the effect of noise. It is recommended that at least in two of 4 experimental conditions we should have x and y be in the same order of *K*_*D*_ (i.e. 0.1 *K*_*D*_ < x, y < 10 *K*_*D*_) to minimize the influence of noise. When we have no prior knowledge about the order of magnitude of *K*_*D*_, we can choose x and y arbitrarily and examine our choice from the histogram of *K*_*D*_. Both the obtained value of *K*_*D*_ and the distribution of the histogram can clearly indicate the appropriateness of our initial choice. Normally in one iteration, we can find the proper range of x, y (i.e. protein and ligand concentration before reaction) that will yield accurate *K*_*D*_.

We have used TIMES signals to find the dissociation constant of protein-ligand interactions from nonmetal-dependent and metal-dependent enzymes: (a) trypsin and *p*-aminobenzamidine (*p*-ABA), (b) thermolysin and phosphoramidon.

Trypsin is a serine protease that hydrolyzes proteins in the vertebrate digestive system^26^. TIMES signals were measured by adding different amounts of *p*-ABA to the trypsin solution (Fig. 3a). What particularly intriguing is that samples with different amounts of *p*-ABA generate TIMES signals that are markedly different from the trypsin signal, indicating that formation of trypsin/*p*-ABA complex can appreciably alter its charge distribution and dipole moment even though *p*-ABA is a much smaller molecule than trypsin. By measuring the TIMES signals produced by different ratios of trypsin and *p*-ABA in the mixtures, we can use Eq. (3) to (a) find the impulse response or Green’s function *q*(*t*) for the protein-ligand complex as well as the reaction dissociation coefficient *K*_*D*_ at each time point, as shown in Fig. 3b. The spikes in the plot of *K*_*D*_ vs time are caused by noise added to Eq. (14). Those spikes are outliers in the histogram plot of *K*_*D*_ in Fig. 3c, and we use the most likely value in the histogram to be the value of *K*_*D*_, which is found to be 34.7 μM. Three repeated runs were performed, and the averaged value agrees with the reported *K*_*D*_ from literatures^27^,^28^ (Table 1). The minor difference could be due to different buffers and pH value in different experiments (we have used 5 mM Tris-HCl buffer at pH = 7.4). In the experiment with another protein ligand pair: thermolysin and phosphoramidon, we have obtained the TIMES signals as shown in Fig. 3d, with *K*_*D*_ calculated in Fig. 3e,f to be 33.1 nM. Repeated experiments showed the averaged *K*_*D*_ value to be 32.1 nM, which is in excellent agreement with the reported values^24^,^29^ (Table 1). The tests were also performed in Hepes buffer and Mops buffer under same temperature and pH, with results shown in Supplementary Materials. The above examples demonstrate that from the TIMES signals, one can investigate protein-ligand binding with a wide range of equilibrium dissociation constants from μMs to nMs. Limited by the noise of transimpedance amplifier, the lowest dissociation constant we can measure with the current setup is ~1 nM.

In summary, we have invented the technique of Transient Induced Molecular Electronic Spectroscopy (TIMES) to detect protein-ligand binding without labeling or immobilization of molecules. The TIMES technique allows us to study the undisturbed interactions between protein and ligand. The method is established on the principle that protein-ligand binding can result in detectable changes in protein’s charge distribution and dipole moment even though the protein-ligand complex has nearly the same molecular weight and chemical makeup as the protein itself. We have mathematically formulated the physics of the signals to make the TIMES technique not only a qualitative tool but also a quantitative method to analyze the protein interactions. Using the TIMES method, we demonstrate measurements of dissociation constant for protein-ligand binding and shear-stress dependent adsorption time for protein and protein/ligand complex. We anticipate that with further development, the TIMES method can be used to study protein folding, binding kinetics, protein-protein interaction, protein-aptamer interaction, and other properties important for drug discovery and protein chemistry.

## Methods

### Device fabrication

**Figure 1:**
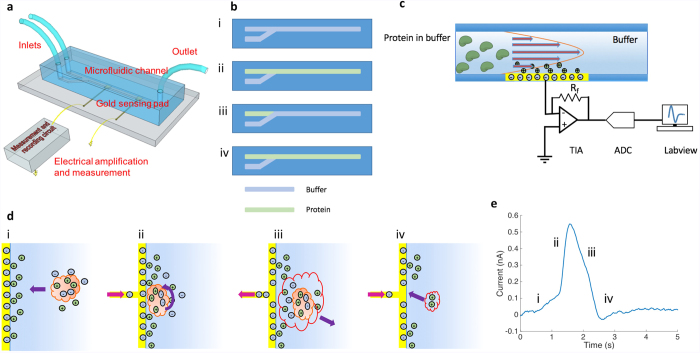
Experimental setup and illustration of the physical process that gives rise to the TIMES signal. (**a**) 3D view of the TIMES setup consisting of a polydimethylsiloxane (PDMS)-based microfluidic channel with gold electrodes and a low-noise transimpedance amplifier circuit. (**b**) The top view of microfluidic channel and the experimental work flow for channel conditioning, protein loading, and testing. (**b**,**i**) Before the experiment, the channel is flushed and filled with buffer injected from both inlets. (**b**,**ii**) One of the inlets is replaced with protein (or other biomolecules of interest) laden buffer to fill the channel. (**b**,**iii**) The syringe pump that drives protein flow is stopped and the other syringe pump driving the buffer is turned on to wash the channel. This procedure completes the conditioning of the system before test, leaving a liquid interface between protein and buffer solutions near the input of the channel. (**b**,**iv**) To start the experiment, the flow of the buffer is stopped and the protein laden solution flows through microfluidic channel. (**c**) Side view schematic of laminar flow in the microfluidic channel, charge distribution at the electrode/liquid interface, and the measurement and recording circuit system. (**d**) Illustration of charge movement, protein reorientation, and signal generation. (**d**,**i**) Local charge neutrality is maintained while a molecule is diffused towards the gold electrode. (**d**,**ii**) Ion redistribution and protein dipole moment reorientation occur when the protein is near the gold electrode. Charge is transferred between the gold electrode and the external circuit due to the image charge effect. (**d**,**iii**) Protein leaves the electrode and drags the local ions via electrostatic interactions. (**d**,**iv**) Ions return to the electrode and the steady state is restored. (**e**) The measured TIMES signal produced by 500 nM thermolysin.

The device was fabricated on a 1 mm thick glass slide. The glass slide was cleaned by sonication in acetone, methanol, and IPA for 5 minutes in each chemical, and blow-dried by nitrogen gas. The glass slide was first lithographically patterned by NR9-1500PY photoresist (Futurrex, USA). After deposition of 100 nm titanium (Ti) and 200 nm gold (Au) films on the glass slide using a sputtering system (Denton Discovery 18, Denton Vacuum, LLC), lift-off process with low power sonication was employed to remove the photoresist to form the Ti/Au patterns. Each Ti/Au sensing area is 1 × 1 mm^2^ with an extended area to allow connection to the external circuits via soldered wires. The microfluidic channel was made of polydimethylsiloxane (PDMS, Sylgard 184, Dow Corning, MI) and patterned using soft lithography (molding) process. SU8-2050 (Microchem) photoresist of 30 μm thickness was patterned on a Si wafer to form the mold for microfluidic channels. Two parts of PDMS (base:curing agent = 10:1) were well mixed and degased in a vacuum chamber, and were poured onto the SU-8 mold and cured at 65 °C for 4 hours. After curing, PDMS was separated from the SU-8 mold and holes were punched to form fluid inlets and outlets. Finally, after UV ozone treatment the PDMS part and the Ti/Au patterned glass slide were aligned under microscope, and baked on 120 °C hotplate for half an hour to ensure bonding, which produced the device in Fig. 1.

### Experiment setup

The two inlets of microfluidic channel were connected to two syringes (BD plastic) that contained buffer and buffer with dissolved protein/ligand. The flow rates of the syringes were controlled by programmable syringe pumps (Pump 11 elite, Harvard Apparatus). One gold sensing area within the microfluidic channel was connected electrically to the input of a low noise transimpedance amplifier (SR570, Stanford Research System, Inc), and the other gold pad within the microfluidic channel was connected to the ground of the circuit. The output of the amplifier was digitized by a DAQ board (National Instrument USB-6251). The data were recorded and filtered using Labview SignalExpress, at a sampling rate of 1 kHz. All the components in the setup, including the stainless steel needles connecting the syringes and the inlets of the microfluidic channels are grounded electrically. Before the experiment, the microfluidic channel is flushed and filled with buffer injected from both inlets (Fig. 1b,i). Then one of the inlets is replaced with protein (or other biomolecules of interest) laden buffer to fill up the channel, as shown in Fig. 1b,ii. Then the syringe pump that drives the protein flow is stopped and the other syringe pump driving the buffer is turned on to wash the channel. The above procedure completes the conditioning of the system before test, leaving a liquid interface between protein and buffer solution near the input of the channel, as shown in Fig. 1b,iii. To start the experiment, the flow of the buffer is stopped and the protein laden solution flows through the microfluidic channel (Fig. 1b,iv). The TIMES signal is generated as shown in Fig. 1c–e.

### Data analysis

After testing protein, ligand, and different ratios of protein and ligand mixture, signals were processed by applying the physical model discussed in the Supplementary Materials to attain such information as induced charge response *q*(*t*), protein (or protein/ligand complex) adsorption time *τ*_*s*_, and dissociation constant *K*_*D*_. The physical model is implemented in Matlab.

## Additional Information

**How to cite this article**: Zhang, T. *et al*. Transient Induced Molecular Electronic Spectroscopy (TIMES) for study of protein-ligand interactions. *Sci. Rep.* **6**, 35570; doi: 10.1038/srep35570 (2016).

## Supplementary Material

Supplementary Information

## Figures and Tables

**Figure 2:**
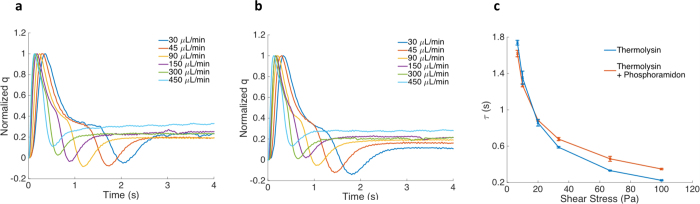
Induced charge response *q*(*t*), and the adsorption time *τ*_*s*_, of a single protein or protein/ligand complex under different flow rates (shear stress) obtained from the TIMES signal using Eq. (2). Induced charge response by thermolysin (**a**) and thermolysin/phosphoramidon complex (**b**) under different flow rate (shear stress) from 30 μL/min (6.67 Pa) to 450 μL/min (100 Pa). (**c**) Shear stress dependent surface adsorption time of thermolysin and thermolysin/phosphoramidon complex.

**Figure 3:**
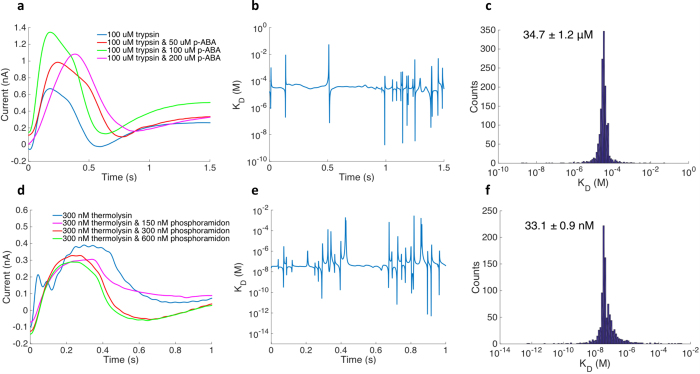
TIMES signals with different ratios of protein ligand concentrations. 5 mM Tris-HCl buffer was used for all the experiments. Solutions with different concentrations of protein and ligand were mixed under room temperature for 3 hours before running the experiment. (**a**) Signals from100 μM trypsin mixed with 0, 50, 100, and 200 μM of *p*-ABA. (**b**) The calculated equilibrium dissociation constant *K*_*D*_ from TIMES signals using Eq. (3). (**c**) Histogram of *K*_*D*_ for trypsin and *p*-ABA reaction from 1000 data points over the 1 s measurement period. The mean value of *K*_*D*_ is also shown in the figure. (**d**) Signals from 300 nM thermolysin mixed with 0, 150, 300, and 600 nM of phosphoramidon. (**e**) The calculated equilibrium dissociation constant *K*_*D*_ from TIMES signals using Eq. (3). (**f**) Histogram of *K*_*D*_ for thermolysin and phosphoramidon reaction from1000 data points over the 1 s measurement period. The mean value of *K*_*D*_ is also shown in the figure.

**Table 1:** Summary of measured dissociation constant from TIMES and literatures.

Protein-ligand pairs	Measured *K*_*D*_ from TIMES	Reported *K*_*D*_ from literatures
Trypsin and *p*-ABA	39.1 ± 3.6 μM	12 uM (Markwardt *et al*.^27^)
		19 uM (Malanikova *et al*.^28^)
Thermolysin and phosphoramidon	32.1 ± 1.9 nM	23 nM (Kitagishi *et al*.^24^)
		28 nM (Komiyama *et al*.^29^)
