# Supplementary material for: Transient Induced Molecular Electronic Spectroscopy (TIMES) for study of protein-ligand interactions
Source: Sci Rep. 2016 Oct 19;6:35570. doi: 10.1038/srep35570 (PMC5069662; doi:10.1038/srep35570)
Supplement: Supplementary Information [file srep35570-s1.pdf]

Supplementary Materials

Transient Induced Molecular Electronic Spectroscopy (TIMES) for study of protein-ligand interactions

Tiantian Zhang[*1], Ti-Hsuan Ku[*2], Yuanyuan Han[2], Ramkumar Subramanian[2], Iftikhar Ahmad Niaz Oni[2], Hua Luo[2,3], Derrick Chang[4], Jian-Jang Huang[5,6], Yu-Hwa Lo[+,1,2]

[1]Materials Science and Engineering Program, University of California San Diego, La Jolla, California 92093-0418, USA.

[2]Department of Electrical and Computer Engineering, University of California San Diego, La Jolla, California 92093-0407, USA.

[3]College of Basic Medicine and Forensic Medicine, College of Manufacturing Science and Engineering, Sichuan University, Chengdu, Sichuan 610041, China.

[4]Department of Nanoengineering, University of California San Diego, La Jolla, California, 92093-0448, USA.

[5]Graduate Institute of Photonics and Optoelectronics, National Taiwan University, Taipei, 10617, Taiwan.

[6]Department of Electrical Engineering, National Taiwan University, Taipei, 10617, Taiwan.

[*]These authors contribute equally to this work.

[+]Correspondence and requests for materials should be addressed to Y-H.L. (ylo@ucsd.edu).

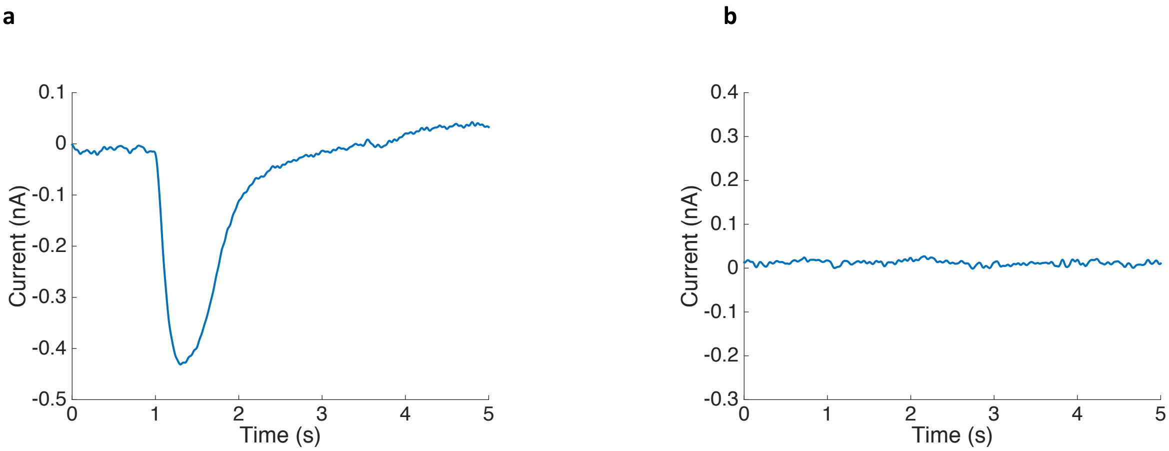

Supplementary Figure S1| Control experiments. (**a**) Signal showing the opposite polarity to the signal in **Fig. 2d** with a reversed protein concentration gradient. To reverse the protein concentration gradient, the channel was first filled with 500nM thermolysin in 5 mM Tris-HCl buffer and then the same buffer without protein was flowed into the microfluidic channel to drive the protein away from the electrode. (**b**) Signal generated by protein-free buffer flowed into the microchannel filled with the same 5 mM Tris-HCl buffer. The absence of detectable signal supports that the observed TIMES signal was produced by protein instead of other side effects.

Unless mentioned otherwise, we have used 5 mM Tris-HCl buffer for all the experiments reported in this paper, including the control experiments described in the following. To show that protein flux from a concentration gradient contributes to the TIMES signal, we

created a reverse concentration gradient for trypsin by first filling the channel with trypsin and then flowing buffer into the channel to drive the trypsin away from the electrode. By reversing the protein concentration gradient, we observed that the polarity of the TIMES signal was also reversed (**Supplementary Fig. S1a**). The result supports our hypothesis and provides the rationale of the experimental design.

We conducted negative control experiment by introducing Tris-HCl buffer into a Tris-HCl buffer filled channel. The absence of any detectable signal (**Supplementary Fig. S1b**) suggests that no signal was produced by artifact such as temperature gradient or flow-induced shear stress.

Supplementary Table S1 | PDB-ID and calculated dipole moment of proteins.

Protein	trypsin	thermolysin	*p*-ABA	phosphoramidon
Molecular Weight (D)	24.4k	34.6k	135	543
Charge (e)	6	-10	2	-2
Dipole (μ /D)	353	547	3.28	3.71

A more complete list of dipole moments of protein can be found in Protein Dipole Moments Server (http://dipole.weizmann.ac.il/) and Pitt Quantum Repository Molecular Database (https://pqr.pitt.edu/).

Supplementary Table S2 | Summary of measured dissociation constant of two protein ligand pairs from TIMES in three different buffers.

Protein-ligand pairs	Buffers	Measured K_D from TIMES
Trypsin and *p*-ABA	Tris	39.1±3.6μM
	Hepes	54.9±7.1μM
	Mops	30.9±4.0μM
Thermolysin and phosphoramidon	Tris	32.1±1.9nM
	Hepes	27.0±2.6nM
	Mops	19.1±1.8nM

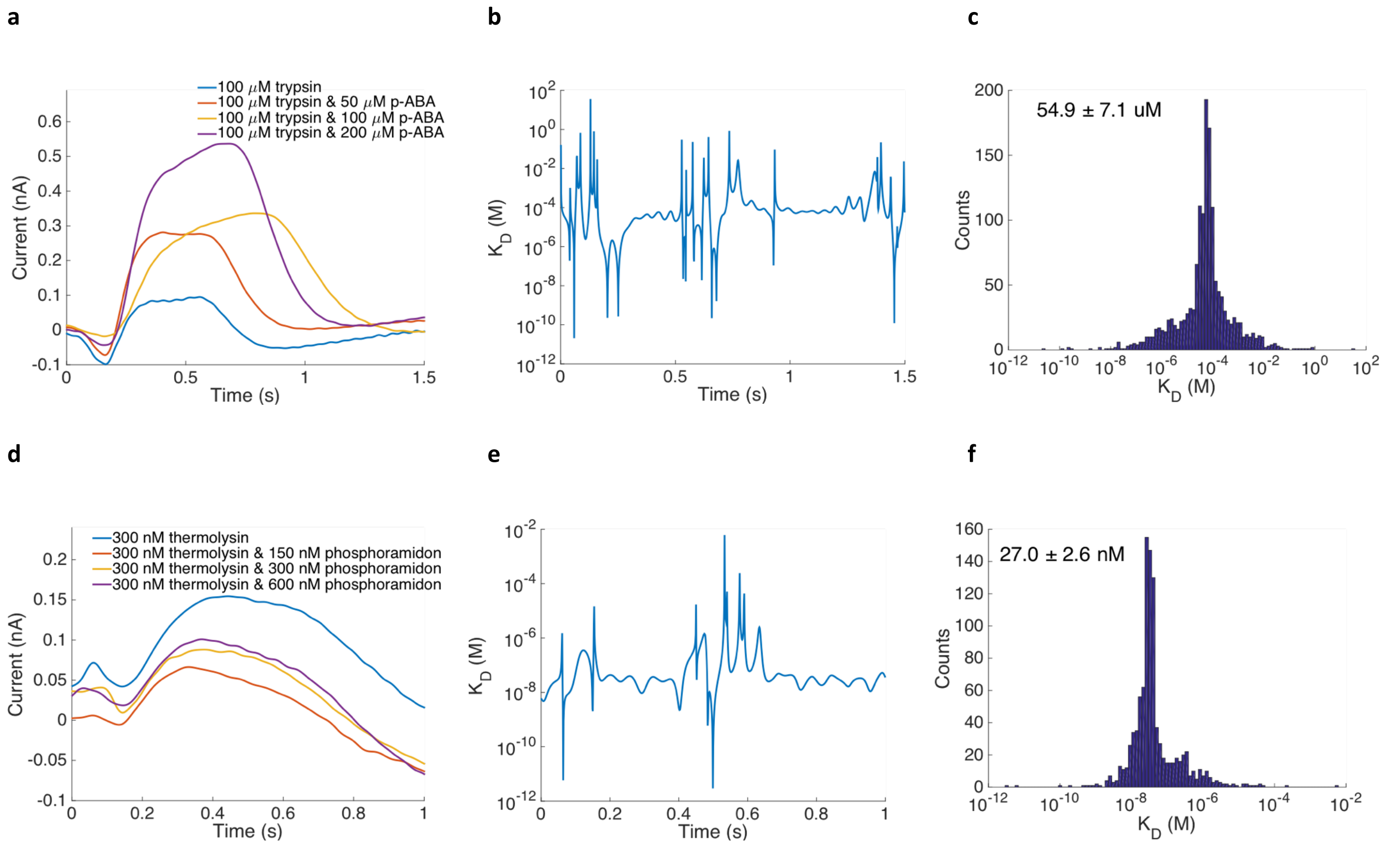

Supplementary Figure S2| TIMES signal in 5mM Hepes buffer. **a-c**. Trypsin and *p*-ABA, with K_D calculated to be 54.9μM. **d-f**. Thermolysin and phosphoramidon, with K_D calculated to be 27.0nM.

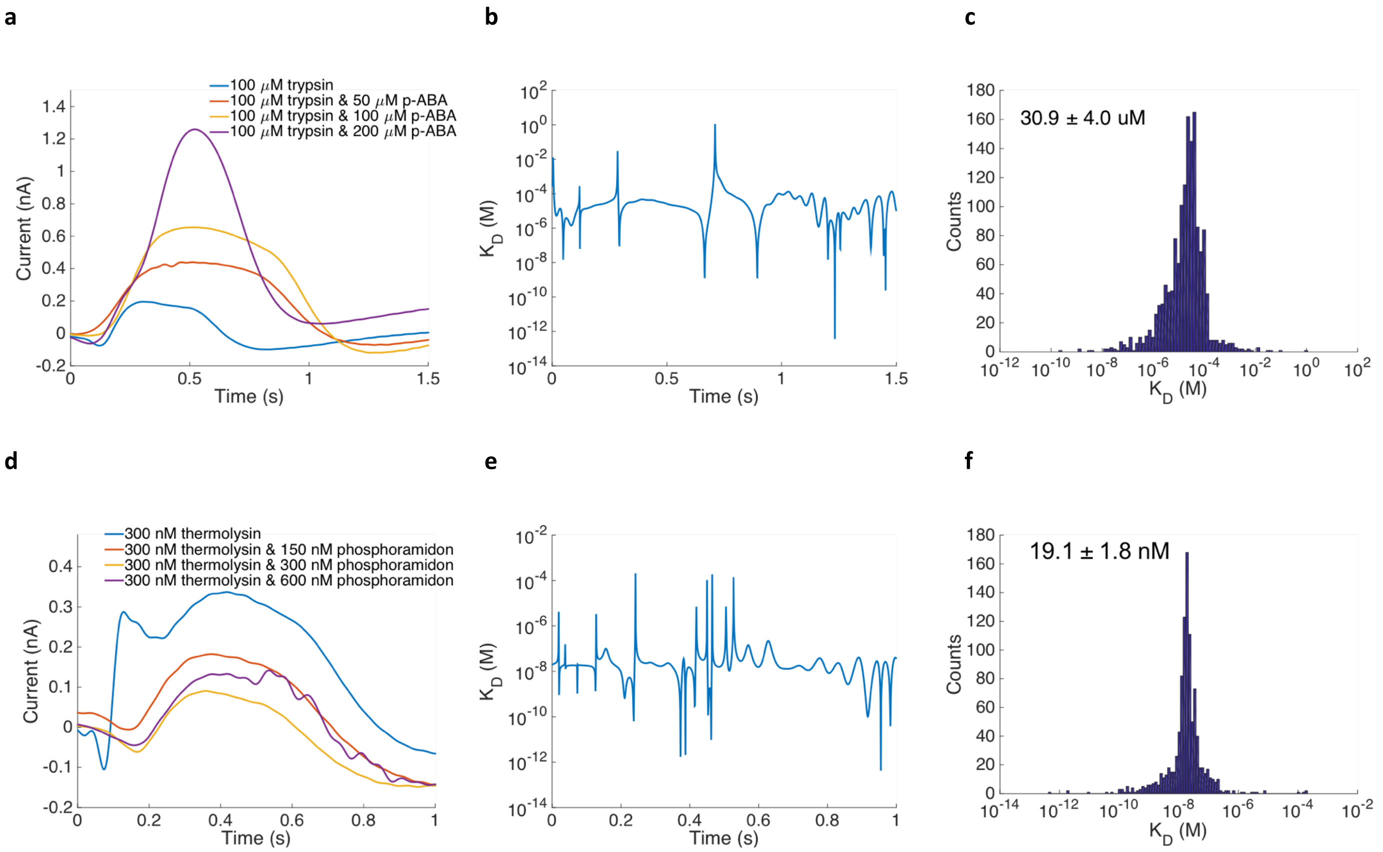

Supplementary Figure S3| TIMES signal in 5mM Mops buffer. **a-c**. Trypsin and *p*-ABA, with K_D calculated to be 30.9μM. **d-f**. Thermolysin and phosphoramidon, with K_D calculated to be 19.1nM.

Physical Model:

Assume that each time a protein hits the electrode surface, it will induce a charge on the gold electrode it contacts: *q(t)*. *q(t)* has the unit of Coul. The protein may stay on the electrode surface or leaves the surface after a time period τ_S.

The net charge signal produced by all the protein molecules at a specific time becomes

$$S(t) = A\int_0^t q(t-u)J(u)du = A\int_0^t q(u)J(t-u)du \qquad (1)$$

where $J(t)$ is the net flux of protein towards the electrode, having the unit: #/Area-s.

Taking the time derivative of (1), we obtain

$$\frac{dS(t)}{dt} = Aq(t)J(0) + A\int_0^t q(u)\left[\frac{d}{dt}J(t-u)\right]du \qquad (2)$$

$\frac{dS(t)}{dt}$ is the amount of protein-induced current (unit: Amp).

We divide the fluid into two regions: region 1 covers the entire space in the microfluidic channel and region 2 is the proximity to the surface of the electrode, typically within the Debye length of the thickness of around 1 nanometer.

Since there exists no electric field in region 1 and because of the laminal flow, the protein concentration away from the electrode interface is governed by the diffusion equation due to protein concentration gradient. We have

$$\frac{\partial n}{\partial t} = D\frac{\partial^2 n}{\partial x^2} \qquad (3)$$

with the boundary conditions:

$n(t, x = L) = n_o$ for $t \geq 0$

$\frac{\partial n(t,x=0)}{\partial x} = 0$ for $t \geq 0$

$n(t = 0, x) = 0$ for $0 \leq x < L$ (4)

As shown in the figures below, the problem in (4) is equivalent to the problem in (5) with different boundary conditions:

$n(t, x = L) = n_o$ for $t \geq 0$

$n(t, x = -L) = n_o$ for $t \geq 0$

$n(t = 0, x) = 0$ for $-L < x < L$ (5)

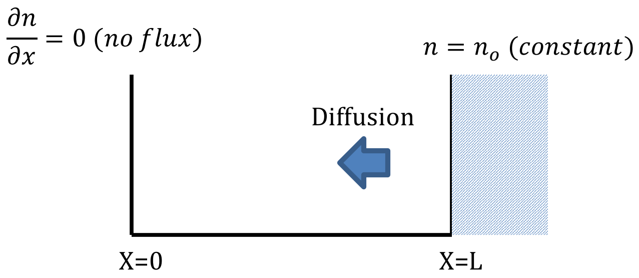

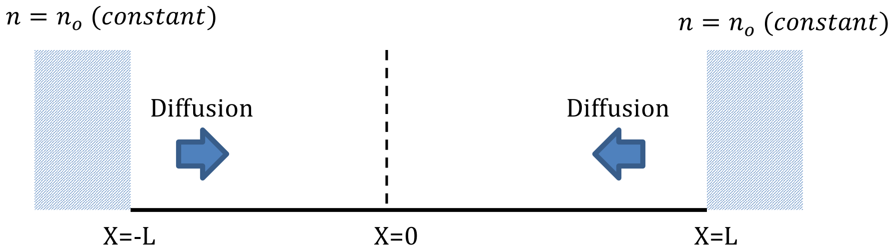

Assume $u(x,t) = T(t)X(x) is\ a\ solution\ for\ (3)$

$$\frac{1}{T(t)}\frac{dT(t)}{dt} = \frac{D}{X(x)}\frac{d^2X(x)}{dx^2} = -k$$

$$u(x,t) = e^{-kt}\left[C_k \cos\left(\sqrt{\frac{k}{D}}x\right) + E_k \sin\left(\sqrt{\frac{k}{D}}x\right)\right]$$

The general solution can be represented as

$$n(x,t) = n_o - \sum_k e^{-kt}\left[C_k \cos\left(\sqrt{\frac{k}{D}}x\right) + E_k \sin\left(\sqrt{\frac{k}{D}}x\right)\right] \qquad (6)$$

Using the boundary conditions in (5), we have

$$\frac{\partial n(x=0,t)}{\partial x} = 0 = \sum_k \sqrt{\frac{k}{D}} e^{-kt}\left[-C_k \sin\left(\sqrt{\frac{k}{D}}x\right) + E_k \cos\left(\sqrt{\frac{k}{D}}x\right)\right]_{x=0} \qquad \text{for } t > 0$$

This means $E_k' s = 0$

$$n(x,t) = n_o - \sum_k e^{-kt}\left[C_k \cos\left(\sqrt{\frac{k}{D}}x\right)\right] \qquad (7)$$

Also from (5) we require

$$n(x = \pm L, t) = n_o - \sum_k e^{-kt}\left[C_k \cos\left(\sqrt{\frac{k}{D}}L\right)\right] = n_o \text{ for } t > 0$$

Then we have $\sqrt{\frac{k}{D}}L = \left(M + \frac{1}{2}\right)\pi \qquad M: -\infty, \ldots . \infty$

$$n(x,t) = n_o - \sum_{M=-\infty}^{\infty} e^{\frac{-(M+1/2)^2 Dt}{L^2}} C_M \cos\left(\frac{(M+\frac{1}{2})\pi x}{L}\right) \text{ for } t \geq 0$$

The above equation can be simplified as

$$n(x,t) = n_o - \sum_{M=0}^{\infty} C_M{}' e^{\frac{-(2M+1)^2 Dt}{4L^2}} cos\left(\frac{(2M+1)\pi x}{2L}\right) \qquad \text{for } t \geq 0 \qquad (8)$$

At t=0,

$$n(x, t = 0) = 0 = n_o - \sum_{M=0}^{\infty} C_M{}' \, cos\left(\frac{(2M+1)\pi x}{2L}\right) \qquad \text{for } -L < x < L \quad (9)$$

$$C_M{}' = \frac{n_o \int_{-L}^{L} cos\left(\frac{(2M+1)\pi x}{2L}\right) dx}{\int_{-L}^{L} cos^2\left(\frac{(2M+1)\pi x}{2L}\right) dx} = \frac{4n_0(-1)^M}{(2M+1)\pi} \qquad (10)$$

Hence

$$n(x,t) = n_o - \sum_{M=0}^{\infty} \frac{4n_o(-1)^M}{(2M+1)\pi} e^{\frac{-(2M+1)^2 Dt}{4L^2}} cos\left(\frac{(2M+1)\pi x}{2L}\right) \qquad (11)$$

We are interested to know $n(x = 0, t)$

$$n(0,t) = n_o - \sum_{M=0}^{\infty} \frac{4n_o(-1)^M}{(2M+1)\pi} e^{\frac{-(2M+1)^2 Dt}{4L^2}} \qquad (12)$$

Note that (12) is the protein concentration in region 1 where there is no electric field. At the surface of the electrode, the protein concentration can be approximated as

$$n_i(t) = n(0,t) e^{\frac{-Ze\zeta}{kT}} = \left[n_o - \sum_{M=0}^{\infty} \frac{4n_o(-1)^M}{(2M+1)\pi} e^{\frac{-(2M+1)^2 Dt}{4L^2}} \right] e^{\frac{-Ze\zeta}{kT}} = \gamma n(0,t) \qquad (13)$$

$$\gamma = e^{\frac{-Ze\zeta}{kT}}$$

where Ze is the charge of the protein and ζ is the zeta potential. We have assumed that the protein concentration is low enough not to change the ionic strength of the buffer. Therefore the zeta potential is not changed significantly by the protein.
